# Interaction of the Retinoblastoma Protein with Orc1 and Its Recruitment to Human Origins of DNA Replication

**DOI:** 10.1371/journal.pone.0013720

**Published:** 2010-11-09

**Authors:** Ramiro Mendoza-Maldonado, Roberta Paolinelli, Laura Galbiati, Sara Giadrossi, Mauro Giacca

**Affiliations:** 1 Molecular Medicine Laboratory, International Centre for Genetic Engineering and Biotechnology (ICGEB), Trieste, Italy; 2 Molecular Biology Laboratory, Scuola Normale Superiore, Pisa, Italy; 3 Department of Biomedicine, University of Trieste, Faculty of Medicine, Trieste, Italy; Texas A&M University, United States of America

## Abstract

**Background:**

The retinoblastoma protein (Rb) is a crucial regulator of cell cycle progression by binding with E2F transcription factor and repressing the expression of a variety of genes required for the G1-S phase transition.

**Methodology/Principal Findings:**

Here we show that Rb and E2F1 directly participate in the control of initiation of DNA replication in human HeLa, U2OS and T98G cells by specifically binding to origins of DNA replication in a cell cycle regulated manner. We show that, both in vitro and inside the cells, the largest subunit of the origin recognition complex (Orc1) specifically binds hypo-phosphorylated Rb and that this interaction is competitive with the binding of Rb to E2F1. The displacement of Rb-bound Orc1 by E2F1 at origins of DNA replication marks the progression of the G1 phase of the cell cycle toward the G1-S border.

**Conclusions/Significance:**

The participation of Rb and E2F1 in the formation of the multiprotein complex that binds origins of DNA replication in mammalian cells appears to represent an effective mechanism to couple the expression of genes required for cell cycle progression to the activation of DNA replication.

## Introduction

Fundamental work initially carried out in S. cerevisiae and then extended to other organisms, has shown that origins of DNA replication are the sites at which the ordered assembly of the multi-protein pre-replicative complex (pre-RC) takes place. During the G1 phase of the cell cycle, the six-subunit origin recognition complex (ORC) is first recruited onto DNA; the CDC6 and Cdt1 proteins are then required together for loading the putative replicative helicase (Mcm2-7) onto chromatin. Initiation of DNA replication occurs through the function of Cdc7 and S-phase CDKs, which activate the pre-RC and promote further recruitment of proteins required for DNA synthesis [1], [2]. In S. cerevisiae, origin selection is essentially dictated by the binding of ORC to a specific, A/T rich, 11-bp DNA sequence. In higher eukaryotes, however, despite the impressive conservation of proteins participating in the pre-RC formation, primary DNA sequence recognition by ORC is loose, and origin selection requires further information beyond ORC-DNA interaction, possibly indicating that other proteins participate in ORC positioning either directly or through the induction of epigenetic modifications of chromatin [3].

The expression of a large number of factors that participate in the formation of the pre-RC, or that are required for the G1-S transition and the progression of the replicative fork, is transcriptionally controlled by the interaction of the E2F1-3 transcription factors with the retinoblastoma (Rb) protein. In early G1 cells, E2F-bound, hypo-phosphorylated Rb inhibits E2F activity through both direct and indirect mechanisms, which include masking the E2F activation domain or recruiting transcriptional repressors and chromatin modifiers [4], [5]. Upon phosphorylation by the G1 phase CDKs, repression by Rb is relieved and transcription of the E2F-target genes ensues. This is, therefore, an effective mechanism to couple cell cycle progression to the expression of genes that are required for DNA replication.

Over the last few years, however, a few observations have indicated that E2F and Rb might also participate more directly in the regulation of DNA replication. Rb was found to affect the spatial organization of DNA replication in primary mammalian cells [6] and to prevent genomic re-replication in cells experiencing S phase DNA damage, G2/M arrest and M phase block [7], [8]. More specifically, in cells irradiated in early S phase, Rb was found associated with an early firing origin of DNA replication (the Lamin B2 origin) during the S-phase block, and then with additional origins in the order in which they fired [9]. Finally, and most notably, studies performed in D. melanogaster have shown that the E2F1 and Rb homologues form a complex with Drosophila ORC and associate with the chorion gene cluster origin of DNA replication, thereby limiting the physiological amplification of this cluster in ovarian follicle cells [10], [11].

Taken collectively, this information strongly suggests that E2F and Rb might be part of the complex of proteins that associate with origins of DNA replication also in vertebrates. Indeed, here we show that both Rb and E2F1 bind three human origins of DNA replication and that this association is strictly regulated during the cell cycle. We describe that, both in vitro and inside the cells, the largest ORC subunit (Orc1) directly interacts with the under-phosphorylated form of Rb, and that this interaction is mutually exclusive with the binding of Rb to E2F1. Consistent with these findings, down-regulation of Orc1 by RNA interference in human cells favors E2F1 recruitment onto origins and induces a marked G1 arrest.

## Results

### Rb and E2F1 proteins are recruited to human origins of DNA replication

We investigated recruitment of E2F and Rb to three human origins of DNA replication that our laboratory has described in molecular detail over the last few years. One of these origins encompasses the 3′ end of the lamin B2 gene and the promoter of the downstream TIMM13 gene in chromosome 19q – Lamin B2 origin [12], [13]; the other two origins (GM-CSF Ori1 and Ori2) are located downstream of the GM-CSF gene in human chromosome 5q [14]. Using a high resolution chromatin immunoprecipitation (ChIP) procedure, we have recently mapped the regions of binding of several components of the pre-RC, including Orc1, Orc2, Mcm5 and CDC6, at these origins in close correspondence to the sites of nascent DNA synthesis [14].

**Figure 1:**
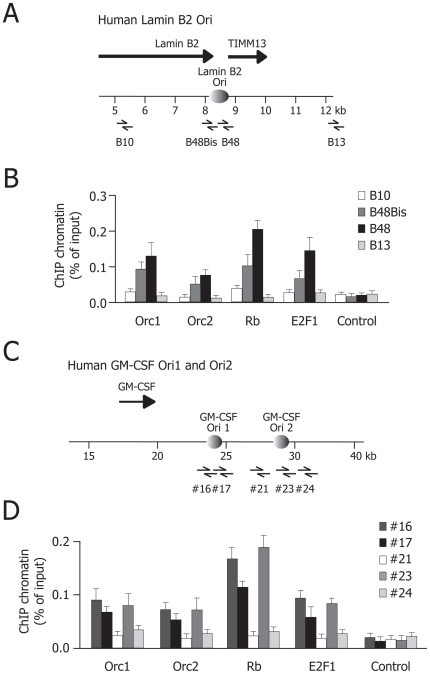
Rb and E2F1 proteins are recruited to human origins of DNA replication. The schemes (**A**) and (**C**) show the genomic regions containing the Lamin B2 origin and the two GM-CSF origins, respectively. Converging arrows indicate sets of primers. The histograms (**B**) and (**D**) show the quantification of crosslinked DNA immunoprecipitated by ChIP on the Lamin B2 and GM-CSF origins, respectively. Each graph shows the specific amplified genomic regions from the origins and the antibodies used for ChiP experiments. The bars indicated as Control show the results obtained by using an irrelevant antibody (normal rabbit IgG). The histograms report the results (mean and standard error of the mean, indicated by error bars) of at least three different experiments. The results are presented as a percentage of the amounts of precipitated chromatin over input DNA. For each of the investigated antibodies, but not for the control, the abundance of the immunoprecipitated regions investigated showed statistically significant difference between origin and non-origin regions (B48 and B48bis for Lamin B2 Ori and #16, #17 and #23 for the GM-CSF oris; *P*<0.05).

Cross-linked chromatin from asynchronous HeLa cells was immunoprecipitated with antibodies against Rb, E2F1, Orc1 and Orc2 proteins. Sequence-specific primer and probe sets for real time PCR analysis were designed to amplify and detect origin-specific regions (B48 and B48bis for the Lamin B2 origin and regions #16, #17 and #23 for the GM-CSF origins), as well as non-origin areas (B10 and B13, flanking the Lamin B2 ori, and #21 and #24 for the two GM-CSF origins); **Figs. 1A and 1C**. These ChIP experiments revealed that, for all the three analyzed origins, origin DNA was enriched 2–5 times over control by immunoprecipitating chromatin with antibodies recognizing Rb and E2F1 (**Figs. 1B and 1D** for Lamin B2 and GMS-CSF origins respectively).

### Orc1 specifically interacts with Rb in vitro and competes with E2F1 for binding

**Figure 2:**
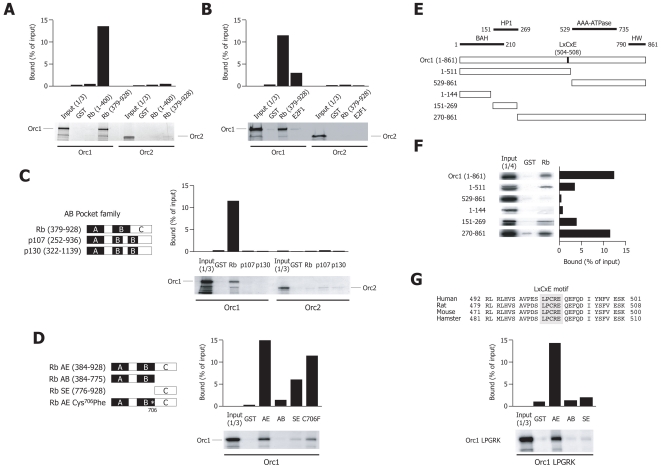
Orc1 specifically interacts with Rb in vitro. (**A**) GST pull-down experiment performed by incubating GST alone or GST fused to the N-terminus (aa. 1–400) or the C-terminus (aa. 379–928) of Rb, immobilized on gluthatione-agarose beads, with in vitro translated [^35^S]-labelled Orc1 or Orc2 proteins. The upper panel shows the quantification of the [^35^S]-labelled protein after in vitro binding. The amount of radioactivity bound to the beads is indicated as a percentage of the input material. The lower panel shows the autoradiography. The Input lanes contain the labelled proteins prior to binding. A representative experiment of at least 3 performed is shown. (**B**) GST pull-down experiment performed by incubating GST, GST-Rb (379–928) or GST-E2F1 fusion proteins on gluthatione-agarose beads with in vitro translated [^35^S]-labelled Orc1 or Orc2. The results are shown as in panel (A). (**C**) Binding to Orc1 is specific for Rb. The scheme on the left side shows the conserved A/B pocket domains in the three members of the RB family, which were used as GST fusion proteins. The figure on the right side, shows the result of a GST pull-down experiment performed with these proteins, and in vitro translated Orc1 and Orc2. “Rb” for short corresponds to the C-terminus of Rb (aa. 379–928). (**D**) Binding to Orc1 requires the C-terminal region of Rb. The scheme on the left side of the figure shows the Rb truncated or mutated proteins used for mapping the domains required for binding to Orc1 (AE, containing the A, B and C pockets; AB, A and B pockets only; SE, C pocket only; AE Cys706Phe, which does not bind LxCxE proteins). These proteins were obtained as GST fusions and used for the GST pull-down experiment shown on the right side of the panel. (**E**) Schematic representation of the main functional domains of the Orc1 protein. BAH, bromo-adjacent homology domain; HP1, HP1 binding domain; AAA, ATPase domain; HW, putative DNA binding site. The fragments of Orc1 subsequently tested by in vitro GST pull-down are indicated by the corresponding amino acids on the left side. (**F**) GST pull-down experiment performed with the Orc1 fragments indicated in (E), labelled by in vitro translation, and challenged to GST or GST-Rb proteins. “Rb” for short corresponds to the C-terminus of Rb (aa. 379–928). (**G**) Sequence alignment showing the conserved LxCxE motif found in the Orc1 subunit of human, rat, mouse and hamster (upper part), and GST pull-down experiment performed with the in vitro translated Orc1 LPGRK protein (mutated in the LxCxE motif of Orc1) and the GST-fusion proteins AE, AB and SE (lower part).

To understand the mechanisms of Rb and E2F1 recruitment onto origin DNA, we initially studied the interactions between the two proteins and different components of the pre-RC by GST-pulldown experiments. We found that [^35^S]-labeled human Orc1 was specifically retained on GST-Rb immobilized on glutathione agarose beads. In particular, Orc1 specifically interacted with the C-terminal (aa. 379–928), but not with the N-terminal (aa. 1–400) half of the protein (**Fig. 2A**). In the same experiments, binding E2F1 to Orc1 or either E2F1 or Rb (379–928) to Orc2 were all negative (**Figs. 2A and 2B**; Coomassie stained gels are shown in **Figure S1**).

The C-terminus of Rb contains the A and B pocket domains, which are common to the other members of the pocket proteins family, p107 and p130 [15], while the additional C domain is specific to Rb (**Fig. 2C**). By using fusion proteins corresponding to the pocket domains of Rb, p107 and p130, we observed that binding to Orc1 was specific for Rb only; in the same experiment, Orc2 binding was negative in all proteins (**Fig. 2C**).

To better characterize the domains in the C-terminal portion of Rb that are responsible for Orc1 binding, we obtained a series of GST fusion proteins carrying the whole Rb C-terminus (fragment AE in **Fig. 2D**), the A and B pocket domains (fragment AB) or only the C-terminus (fragment SE). These proteins were tested for binding to radiolabeled Orc1. We found that the integrity of the C-terminal domain of Rb was essential for binding to Orc1; indeed, the Rb C-terminus alone also retained partial capacity to bind Orc1 (**Fig. 2D**). These results are in agreement with the observation that binding to Orc1 is specific to Rb but not to other pocket proteins.

We then sought to identify the Orc1 protein motifs responsible for the association with Rb. Orc1 is known to have a modular structure, conserved in most species, that includes a BAH and an ATPase domain located at the N- and C-terminal regions of the protein, respectively; a region interacting with the HP1 protein partially overlaps with the BAH motif [16]. We constructed a series of Orc1 mutants carrying various deletions in these domains, as schematically shown in **Fig. 2E**, and obtained them as labeled proteins. Neither the N-terminus (aa 1–144) nor the C-terminus (aa 529–861) of Orc1 alone were sufficient for binding. Fragment 270–861 was capable of binding to Rb to the same extent as the full-length protein, suggesting that an interacting domain was between aa 270 and 528. Since fragment 151–269 still showed residual binding, residues before aa 270 are also likely to contribute to binding.

Different cellular proteins are known to bind Rb through a conserved **L**x**C**x**E** motif [17]. Intriguingly, Orc1 displays such a motif starting at position 504 (**L**P**C**R**E**), a sequence that is absolutely conserved in mammals (**Fig. 2G**). This amino acid stretch by itself, however, is not involved in Rb binding, since an Orc1 protein mutated at this sequence (**L**P**G**R**K**) was still capable of binding the whole Rb C-terminus (AE domain in Fig. **2G**). Binding of this mutant, however, was impaired for the C-terminus SE fragment, possibly suggesting that LxCxE region, albeit not essential, might still participate in binding. The cysteine at position 706 of Rb was reported to be essential for binding to LxCxE motif-containing proteins such as the SV40 large T antigen [18], [19], possibly due to the destabilization of the Rb C-terminus [19]. An Rb C706F mutant, however, was still found capable of binding Orc1 (**Fig. 2D**).

**Figure 3:**
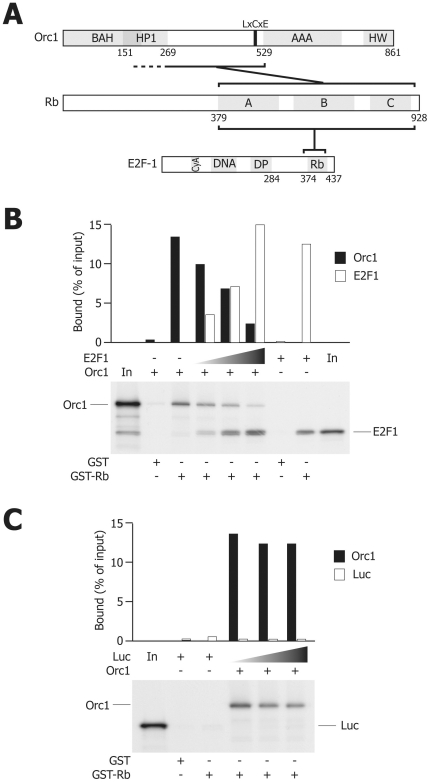
E2F1 competes with Orc1 for binding to Rb. (**A**) Schematic representation of the regions involved in the association of Rb to Orc1 and of those that are necessary for stable association with E2F1. (**B**) GST pull-down experiment performed by incubating a fixed amount of in vitro translated Orc1 together with scalar amounts of in vitro translated E2F1 with an immobilized GST fusion protein containing the ABC pocket of Rb. The graph shows the quantification of bound E2F1 and Orc1 radioactivity; the input lanes (In) contain the labelled proteins prior to binding. (**C**) Competitive GST pull-down control experiment performed with in vitro translated Orc1 and luciferase (Luc) proteins using identical experimental conditions as in (B).

The observation that the integrity of the Rb C-terminus was required for Orc1 binding raised the intriguing possibility that the Rb/Orc1 interaction might be mutually exclusive the binding of Rb to E2F1, which also occurs in the same region of Rb [20] (**Fig. 3A**). To test this possibility, competitive GST pulldown experiments were performed by incubating the recombinant GST-Rb (379–928) fusion protein with in vitro translated Orc1 in the presence of increasing amounts of in vitro translated E2F1. As shown in **Fig. 3B**, E2F1 was found to compete with Orc1 for binding to GST-Rb. In contrast, when a control luciferase protein was used to substitute E2F1, binding of Orc1 to Rb was unaffected (**Fig. 3C**). Collectively, these in vitro results indicate that binding Orc1 i) is specific for Rb and not for other pocket family members; ii) requires the integrity of the Rb C-terminal domain; iii) involves the central portion of Orc1; iv) occurs in an LxCxE motif-independent manner; v) is mutually exclusive with the binding of Rb to E2F1.

### Endogenous Orc1 forms a stable complex with hypo-phosphorylated Rb in human cells

**Figure 4:**
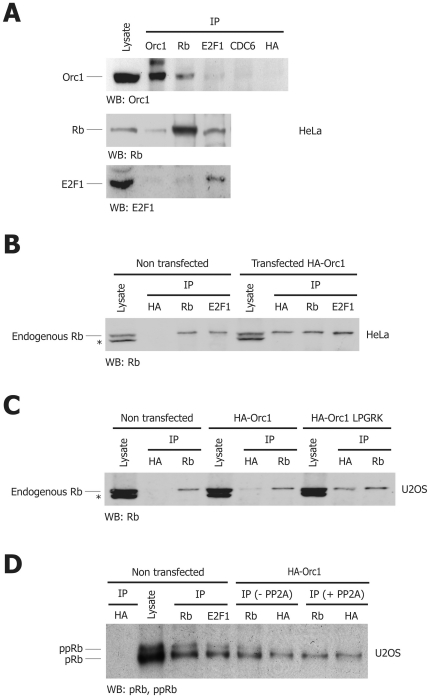
Orc1 forms a stable complex with hypo-phosphorylated Rb inside the cells. (**A**) Co-immunoprecipitation experiments performed with lysates from asynchronous HeLa cells using the indicated antibodies for immunoprecipitation and western blottings. (**B**) Immunodetection of endogenous Rb after co-immunoprecitation with exogenous HA-tagged Orc1 in transiently transfected asynchronous HeLa cells. Additional co-immunoprecipitations with Rb and E2F1 proteins in non-transfected and HA-Orc1-transfected HeLa cells were performed as controls. The band marked by an asterisk (*) represents an unspecific band detected with the mouse anti-Rb antibody IF8. (**C**) Endogenous Rb detected by western blotting after immunoprecipitation with anti-HA peptide antibody in non-transfected, wt HA-Orc1-, and mutant HA-Orc1 LPGRK-transfected U2OS cells. Additional immunoprecipitations for Rb were performed as controls on the same lysates. The band marked by an asterisk (*) represents an unspecific band detected with the mouse anti-Rb antibody IF8. (**D**) Immunoblotting to visualize the differently phosphorylated forms of endogenous Rb, after immunoprecipitation with anti-Rb and anti-HA peptide antibodies in U2OS cells not transfected or transfected with wt HA-Orc1, as indicated. Orc1 immunoprecipitated hypo-phoshorylated Rb, showing the same apparent mass as that obtained after treatment of total Rb immunoprecipitates with PP2A phosphatase.

We proceeded to test the interaction of Orc1 and Rb inside HeLa cells by co-immunoprecipitation experiments, and found that endogenous Orc1 interacted with endogenous Rb, but not with E2F1. Consistent with the results in vitro, we also observed that both endogenous E2F1 and Orc1 co-immunoprecipitated with Rb, and that only Rb co-immunoprecipitated with E2F1 (**Fig. 4A**). These results were further confirmed by transfecting an HA-tagged version of Orc1 in HeLa cells, followed by immunoprecipitation with an anti-HA antibody. In these conditions, endogenous Rb co-immunoprecipitated with HA-Orc1, in addition to endogenous E2F1 (**Fig. 4B**). The same result was also obtained in U2OS cells (**Fig. 4C**). In addition, we tested the interaction of Rb with an HA-tagged protein corresponding to Orc1 mutated in the LxCxE domain (HA-Orc1 LPGRK). Consistent with the GST pulldown experiments, we found that endogenous Rb still bound this mutant (**Fig. 4C**). Finally, we observed that Orc1 preferentially associated with a molecular species of Rb with an apparent molecular mass identical to the hypo-phosphorylated form of the protein (**Fig. 4D**). Collectively, these results indicate that both endogenous and transfected Orc1 form a complex with Rb in different cell types independent of the LxCxE motif, that the formation of this complex is mutually exclusive with the binding of Rb to E2F1, and that it involves the hypo-phosphorylated forms of Rb.

### Visualization of Orc1-Rb interaction inside the cells by fluorescence resonance energy transfer (FRET)

**Figure 5:**
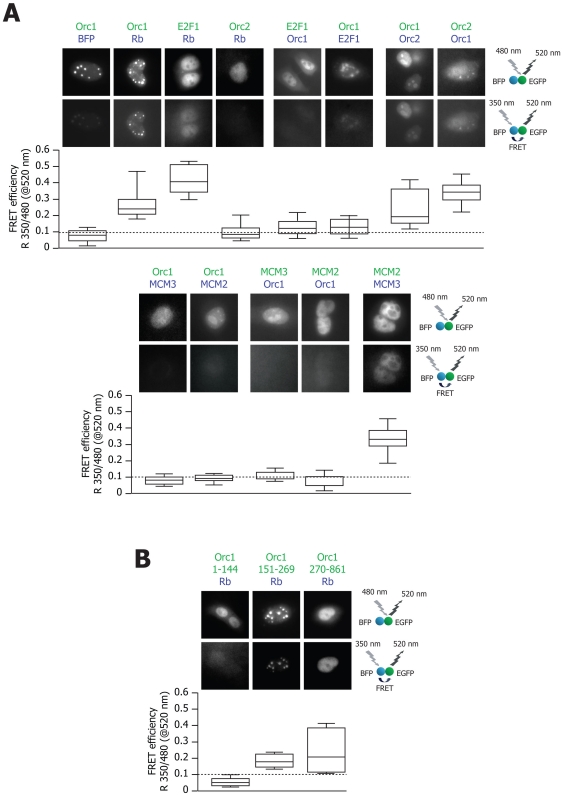
FRET analysis. (**A**) HeLa cells were transiently transfected with expression vectors coding for the proteins indicated on top of each panel fused to either EGFP (green color) or BFP (blue color). Individual transfected cells were visualized by excitation at 480 nm and collection at 520 nm, showing EGFP fluorescence after direct EGFP excitation (panels in the upper row), and by excitation at 350 nm and collection at 520 nm, showing EGFP fluorescence after BFP excitation, indicating FRET (panels in the lower row). The box plot below each image pair shows the quantification of FRET. Fluorescent emission at 520 nm from individual cells was recorded after excitation at 350 or 480 nm, and integrated intensities over the whole cell were evaluated. The percentile box-plot distribution of the ratio between these two measurements is shown by considering at least 10 consecutively analyzed cells for each protein pair. Horizontal lines of the percentile box plot distribution, from top to bottom, mark the 10th, 25th, 50th, 75th, and 90th percentile respectively. (**B**) FRET between Rb, tagged with BFP, and the same set of Orc1 truncation mutants considered for the GST pulldown experiments, tagged with EGFP.

The presence of FRET between two proteins tagged with optically matched pairs of fluorophores indicates direct protein-protein interaction at distances to the order of a nanometer in vivo (see [21] and references therein). FRET experiments were performed by transfection with plasmids expressing different pairs of proteins bearing the EGFP and BFP proteins fused to their N-terminus. FRET image analysis of individual cells is shown in **Fig. 5A**. For each protein pair, the upper panels show the intracellular distribution of fluorescence at 520 nm (the peak wavelength of EGFP emission) under excitation at 480 nm; the lower panels show the fluorescence of the same fields at 520 nm after excitation of BFP at 350 nm. For each protein pair, quantitative analysis of the intensity of fluorescence of at least 10 cells was performed under the two illumination conditions. These results are presented in the box plots below the individual Figures, indicating the percentile distributions of FRET efficiency. These experiments clearly revealed that Orc1 and Rb physically interacted inside the cell's nucleus. Other positive interactions were detected between Rb and E2F1, but not between Rb and Orc2, between Orc1 and E2F, or between Orc1 and either MCM2 or MCM3. Notably, as expected, Orc1 was found to bind Orc2, [16], [22], as well as MCM2 to MCM3.

We also exploited FRET to visualize and quantify the binding of Rb with the same set of Orc1 truncation mutants used in the GST pulldown experiments. We found that EGFP-Orc1 (1–144) was negative for FRET with BFP-Rb; in contrast, clear positivity was detected for both the C-terminal fragment of Orc1 (270–861) and the middle fragment (151–269); **Fig. 5B**. These results provide an in vivo confirmation that the Orc1 fragment 270–861 is capable of binding to Rb to the same extent as the full length protein, and that binding is also enhanced by residues extending before amino acid 270.

### Orc1 and E2F1 are recruited to the lamin B2 origin at different temporal windows of G1 phase

The finding that Rb and E2F associate with origins of DNA replication raises the possibility that these proteins might directly regulate origin function. To start addressing this issue, we analyzed whether the recruitment of Rb and E2F at origins vary during the cell cycle.

**Figure 6:**
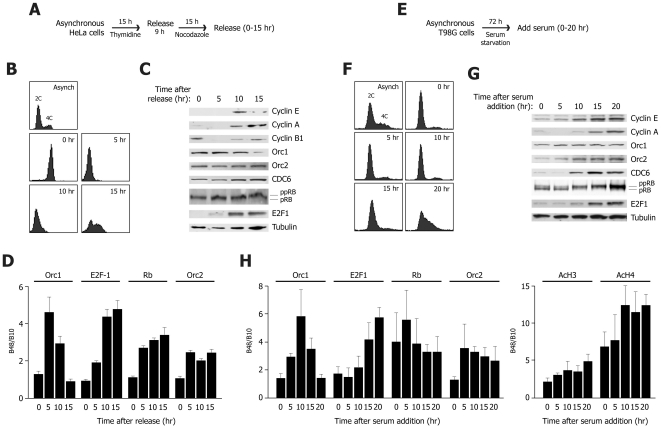
Cell cycle-dependent association of Rb and E2F1 with the Lamin B2 origin. (**A**) Experimental scheme for HeLa cell synchronization. HeLa cells were synchronized in mitosis by a double thymidine/nocodazole block, and then followed G1 after release from the block. (**B**) Flow cytometry profiles of asynchronous cells (Asynch), cells blocked in mitosis (0 hr) or cells at different times after release. (**C**) Western blot analysis of whole-cell extracts obtained from cells at different time points during synchronization. (**D**) Quantification of cross-linked lamin B2 origin DNA immunoprecipitated by ChIP. On top of the graph, the antibodies used for ChIP are shown. The histogram reports the results (mean±sem) of at least three independent experiments. The results are presented as the fold enrichment of the lamin B2 origin region (B48) over the irrelevant B10 region, after normalization for the levels of immunoprecipitated chromatin using an unrelated antibody (normal rabbit IgG) as control. (**E**) Experimental scheme for T98G cell synchronization. Cells were cultured without serum for 72 h and then followed for 20 h after addition of serum. (**F**) Flow cytometry profiles of asynchronous cells (Asynch), cells blocked in G0 by serum starvation (0 hr) or cells at different times after serum stimulation. (**G**) Western blot analysis of whole-cell extracts obtained from cells at different times points during synchronization. (**H**) Results of ChIP experiments for the lamin B2 origin, using the antibodies indicated on top of each dataset. The results are presented as in (D). AcH3: acetylated histone H3; AcH4: acetylated histone H4.

HeLa cells were synchronized in mitosis by sequential treatment with thymidine and nocodazole, and then released from the block and harvested at different times (**Fig. 6A**). The effectiveness of the synchronization treatment to arrest cells in the M phase (0 h) was confirmed by both their 4C DNA content and by the expression of cyclin B1 (**Figs. 6B and 6C** respectively). At 5 h from release, cells had an early G1 profile, characterized by a 2C DNA content. At 10 h, cells were in proximity to the G1/S border, expressing both cyclin E and A. Finally, at 15 h most cells had entered S phase and were characterized by high level expression of cyclin A. Orc1 was found expressed at high levels at 0, 5, and 10 h from mitotic block release; E2F1 started to be present at 5 h and its levels increased at 10 and 15 h; hypo-phosphorylated Rb was detected at 0 and 5 h, while its phosphorylated forms were mainly apparent at 10 and 15 h; Orc2 and CDC6 were present throughout all time points (**Fig. 6C**).

The association of Rb, E2F1, Orc1 and Orc2 proteins with the lamin B2 origin was analyzed by ChIP (**Fig. 6D**). In M-phase cells (0 h), no significant enrichment was found for any of these factors compared to chromatin immunoprecipitated with an irrelevant antibody. Strikingly, Orc1 was found to bind the origin at 5 h (∼5-fold increase over control), and then to be progressively released from it at 10 h (G1/S; ∼3 fold increase), to return to background levels at 15 h (S phase). In contrast, E2F1 showed reciprocal behavior, namely commencing association with the origin as soon as it was expressed (5 h; ∼2-fold enrichment over background), while its binding to the origin increased at 10 and 15 h (G1/S and S respectively; ∼4–5 fold enrichment). Orc2 and Rb were constantly found associated with the origin DNA at all time points after nocodazole block release (∼2–3 fold enrichment).

To better address the study of the kinetics of E2F/Rb recruitment to the lamin B2 origin during the G1 phase of the cell cycle, as well as to investigate factor binding in the G0 phase, we took advantage of the possibility to synchronize human T98G cells in G0 by serum starvation for 72 h [23], [24] (**Fig. 6E**). Upon re-addition of serum, cells synchronously progressed throughout G1 (**Fig. 6F**). In accordance with recently published data [24], cyclin E and cyclin A levels started to rise in middle G1 (10 h) and late G1 (15 hr) respectively, with both cyclins being present at G1/S (20 h). CDC6 started to appear in middle G1; Orc1 and Orc2 were present throughout the synchronization process, even if the levels of the latter protein increased after middle G1. E2F1 progressively increased after middle G1, concomitant with the appearance of hyper-phosphorylated pRb (**Fig. 6G**).

Immunoprecipitations were performed with chromatin crosslinked at the different time points (**Fig. 6H**). In G0 cells, the only antibody that gave significant enrichment on the lamin B2 origin DNA was the one against Rb (∼4-fold over background); binding of Rb to the origin remained constant at all subsequent time points. Similarly, Orc2 started to be detected (∼3-fold enrichment) in early G1 and remained rather constantly bound to the origin. Of interest, Orc1 binding to the origin was not detectable in G0, despite the protein being expressed; in early G1, the protein started to associate with the origin and its enrichment peaked in mid G1 (4–5 fold). At the later time point (15 h) it progressively decreased to become unapparent in cells at G1/S. In contrast, E2F1 showed opposite behaviour, since it started to associate to the origin as early as its levels rose in G1 (∼2-fold enrichment over background in middle G1, ∼4-fold in late G1, over 5-fold in late G1). Parallel to the recruitment of E2F1 to the origin region, and to the detachment of Orc1, the levels of chromatin acetylation, detected using antibodies against acetylated histones H3 and H4, were found to progressively increase over time.

Collectively, these ChIP data are concordant in showing a reciprocal behavior of Orc1 and E2F1 recruitment onto origin DNA. In early G1 cells, the origin is engaged in binding Rb, Orc2 and Orc1; in agreement with the G1/S boundary, Orc1 appears to leave the complex and to be replaced by E2F1. This event parallels the progressive acetylation of chromatin at the origin area. Orc1 detachment from origins in correspondence of the G1-S transition has also been recently reported by Thangavel and coworkers [25].

### Downregulation of Orc1 blocks cells in G1 and increases binding of E2F-1 to origin DNA

**Figure 7:**
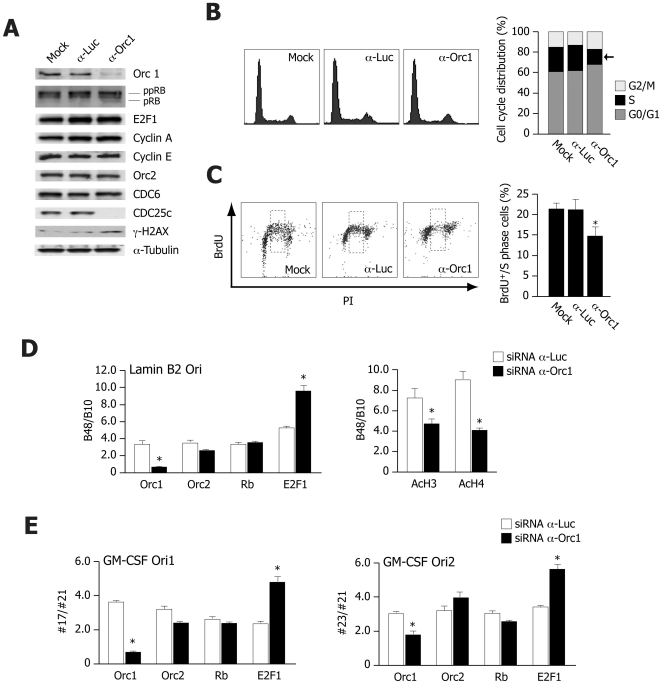
Down-regulation of Orc1 inhibits DNA replication and enhances E2F1 recruitment to origins of DNA replication. (**A**) Western blotting using the indicated antibodies at 72 h after treatment of U2OS cells with siRNAs against Orc1 or luciferase (Luc) control. (**B**) Flow cytometry profiles of U2OS cells treated for 72 h with the indicated siRNAs. The histogram on the right side shows the distribution of the cells in the different phases of the cell cycle; the reduction in the number of S-phase cells after Orc1 silencing is indicated by an arrow. (**C**) Flow cytometry profiles simultaneously showing detection of DNA content (propidium iodide staining) and BrdU incorporation (anti-BrdU antibody) at 72 h after RNAi. The dashed boxes indicate BrdU positive, S-phase cells. The histogram on the right side reports the percentage of S-phase/BrdU positive cells (mean±sem, indicated by error bars) of three different experiments. The asterisk (*) indicates significant statistical difference between ORC depletion experiments and luciferase control experiments. (**D**) Results of ChIP experiments performed in U2OS cells at 72 h after siRNA silencing of Orc1. The histograms show the quantification of origin-specific, cross-linked and immunoprecipitated DNA for the Lamin B2 origin after immunoprecipitation using the antibodies shown below each bar pair. The results are expressed as fold of enrichment of the specific origin sequence (B48) over a neighbouring control sequence (B10), as shown in **Fig. 1A**. The means±sem of at least three different experiments are shown. The asterisk (*) indicates statistically significant differences between ORC depletion experiments and luciferase control experiments. (**E**) Results of ChIP experiments performed by analyzing protein binding to the GM-CSF Ori1 and Ori2 origins in U2OS cells at 72 h after siRNA silencing of Orc1. The results are expressed as in (D), by showing the fold of enrichment of the specific origin sequences (#17 and #23) over a neighbouring control sequence (#21), as schematically represented in **Fig. 1C**.

Next we sought to determine whether depletion of the Orc1 protein could influence the recruitment of Orc2 (as representative of the ORC core complex [26]) or Rb/E2F1 at origins of DNA replication. Orc1 depletion was achieved by RNA interference on U2OS cells. **Fig. 7A** shows that, despite a marked reduction (>80%) of Orc1 protein, Orc2, Cdc6, E2F1, Rb and cyclin A and E protein levels did not vary significantly. Interestingly, no CDC25c protein was detectable in the Orc1 siRNA-treated cells, and expression of the phosphorylated form of histone H2AX was increased; both observations are consistent with the induction of a damage checkpoint as a consequence of the Orc1 knock down.

Orc1 depletion determined a remarkable reduction in the number of cells in the S phase of the cell cycle (from 24% to 15% of total), and a consequent increase in the number of cells in G1 (from 61% to 68%) (**Fig. 7B**). Treatment with an anti-luciferase siRNA control did not modify the cell cycle profiles. To better document the reduction in the number of S-phase cells upon treatment with the anti-Orc1 siRNA, we analyzed DNA synthesis after a 1 h pulse of the siRNA-treated cells with BrdU, followed by flow cytometry using an anti-BrdU antibody. As shown in **Fig. 7C**, the number of cells with a DNA content between 2C and 4C that incorporated BrdU (S-phase cells) was reduced from 21.5% to 14.7%, thus indicating that the consequence of the Orc1 knock down is a marked inhibition of DNA synthesis. This inhibition was even more pronounced in the Rb-null Saos-2 cells (from 18.1% to 7.9% cells in S-phase after Orc1 siRNA treatment; data not shown).

In the siRNA treated cells, we studied the recruitment of Orc1, Orc2, Rb and E2F1 to the lamin B2 origin. We found that the binding of Orc2 and Rb was not significantly affected by the Orc1 knock down. In contrast, Orc1 depletion induced a marked increase in E2F1 binding, as well a significant decrease of the extent of chromatin acetylation at the origin region (**Fig. 7D**). Very similar findings in terms of E2F1 binding were also detected by analyzing the immunoprecipitated chromatin with primer pairs detecting the two origins in the GM-CSF gene domain (**Fig. 7E**).

## Discussion

The work presented in this manuscript presents evidence that both Rb and E2F are part of the protein complex that is recruited to origins of DNA replication at the G1 phase of the cell cycle. This observation raises a series of obvious issues, including the identification of the molecular determinants responsible for their recruitment, the understanding of the relationship of these proteins with the other components of the pre-RC and, most notably, the definition of their actual function in regulating origin activity.

As shown by the immunoprecipitation experiments, Rb and E2F1 are recruited to the lamin B2 origin as well as to the two investigated origins downstream of the human GM-CSF gene; enrichment for the two factors is detected at the same location where other components of the pre-RC are found and are in close correspondence to the sites of nascent strand DNA synthesis [14]. These three origins are rather dissimilar in both primary sequence and chromatin context. In particular, the lamin B2 origin encompasses a promoter and is located in a region that is transcribed at high levels and in a constitutive manner [27]. In contrast, GM-CSF Ori1 and GM-CSF Ori2 are located ∼7 Kb downstream of the GM-CSF gene, in a region that shows no apparent transcription or canonical marks of the presence of cis-acting transcriptional regulation elements. In addition, the three origins show neither obvious primary sequence similarity nor the presence of canonical E2F binding sites. Given these considerations, our first speculation has been that binding Rb/E2F to the origin region might be mediated through the interaction of either protein with known components of the pre-RC. Indeed, our results support the model in which Rb is bound near ORC at origins of DNA replication by forming a specific complex with the largest ORC subunit, Orc1. In particular, the in vitro data that collectively support the specificity of this novel interaction indicate that the binding of Orc1 i) is specific for Rb and not for other pocket family members, ii) requires the integrity of the C-terminus of Rb, iii) involves the central portion of Orc1; and iv) occurs in an LxCxE motif-independent manner. In addition, the in vivo data show physical interaction between Orc1 and Rb inside the nucleus (FRET) and preferential binding of Orc1 to the hypo-phosphorylated form of Rb (co-immunoprecipitations). Recent information indicates that the Drosophila homologue of Rb (Rbf1) can also associate with the Drosophila ORC complex and chromatin [28]. In Drosophila, however, at difference with human Rb, binding to ORC appears to depend on the Rb N-terminus and not on the E2F-binding region.

The recruitment of Rb to origins during the normal cell cycle occurs at a time point at which Orc1 is not yet bound (such as at 5 h after entry into G1 phase after stimulation of serum-starved T98G cells) or in conditions in which Orc1 is knocked down. Thus, binding of Rb to the origin area definitely precedes, and is thus independent from, binding of Orc1, and therefore, the assembly of the pre-RC; what the determinants of Rb recruitment might be, therefore, still remain elusive. In this respect, it is worth mentioning that Rb has been shown to localize to multiple discrete DNA foci during S phase [7] and that its docking to chromatin might thus be dependent on its interaction with other cellular factors. Among the over 100 factors other than E2F that specifically bind Rb [29] there are other proteins that participate in the DNA replication process, including MCM7, MCM4, DNA polymerase alpha and replication factor C [19], [30], [31], [32], [33]). Binding to these factors, however, is likely to occur at a later stage during the origin activation process.

As far as E2F1 is concerned, our in vitro competition GST-pulldown assays and our in vivo ChIP data along G1 phase progression and after Orc1 knock down indicate a mutually exclusive binding of Rb to either Orc1 (early G1) or E2F1 (late G1 and S phase). Indeed, the integrity of the whole Rb C-terminal region, and, in particular, of the C domain, is required for both binding to Orc1 (our findings) as well as to E2F1 [20]. The finding that E2F1 displaces Orc1 from origin-bound Rb during late G1 is consistent with the consolidated notion that Orc1 leaves chromatin in late G1 and during S-phase [34]. Intriguingly, both Rb and E2F1 have been found associated with DNA replication foci in primary cells during early S-phase, a time point at which the canonical regulation of Rb by G1-phase CDKs would instead predict its dissociation from the transcription factor [35]. Indeed, this finding is perfectly consistent with our ChIP data on origin DNA. On this basis, we propose that the primary determinant of E2F binding to the origin regions might not be primary DNA sequence recognition by E2F itself, but its interaction with chromatin-bound Rb, consistent with the information that chromatin-bound Rb is also detected during the S-phase [36].

The finding that Rb and E2F1 associate with origins of DNA replication raises the obvious possibility that these proteins might directly regulate some aspects of origin function. The kinetics of recruitment of the different factors onto the origin region, Rb and Orc1 during early G1 phase, Rb and E2F1 during late G1, along with other pre-RC components [37]; the preferential binding of Orc1 to the hypo-phosphorylated form of Rb; and, finally, the increase in chromatin acetylation that is detected when Orc1 detaches from the origin during the cell cycle or its decrease when Orc1 is downregulated by RNAi, all suggest that the complex of Orc1 with hypophosphorylated Rb negatively regulates origin function. This conclusion is consistent with the observation that mutations in the Drosophila Rb (as well as E2F) homologues fail to limit DNA replication through their interactions with DmORC [11]. This negative function might be exerted by a variety of mechanisms, which recapitulate the known properties of hypophosphorylated Rb, including the modulation of chromatin conformation, or the direct negative regulation on components of the replication licensing machinery.

In conclusion, our results show that Rb participates in the formation of the protein complex that regulates DNA replication origins during the normal cell cycle in mammalian cells, in addition to being a major component of the intra-S-phase checkpoint response after γ-irradiation [9]. The observations that Rb is essential for a proper spatial organization of DNA replication in mammalian cells [6], that primary cells approaching senescence undergo pRB-dependent, large-scale changes in chromatin structure [38], and that cell cycle exit and terminal differentiation are mediated by Rb [39], all raise the important question whether the actual mechanisms mediating some of these effects might be the specific suppression of initiation of DNA replication at origins.

## Materials and Methods

### Cell Culture, cell synchronization and cell cycle analysis

HeLa, T98G, U2-0S and Saos-2 cell lines (all from the ATTC - http://www.atcc.org/) were maintained in Dulbecco's modified Eagle's Medium (DMEM) with Glutamax (Life Tecnologies, Inc.) supplemented with 10% fetal bovine serum (Life Tecnologies, Inc.). HeLa cells were synchronized in M phase by sequential treatment with 2.5 mM thymidine (Sigma) for 15 h, washed and released in fresh medium for 9 h and, finally, blocked with 50 ng/ml nocodazole (Sigma) for 15 h. For the subsequent synchronization through the G1 to the S phase, mitotic HeLa cells were shaken-off, washed and released in fresh medium at different times. T98G cells were synchronized by serum starvation as described previously [40], [23]. Cells were analyzed for cell cycle profile (DNA content) by incorporation of propidium iodide (Sigma) and analyzed by flow cytometry on a FACSCalibur (Becton Dickinson). Cell cycle profile distributions were determined with the Modfit LT 3.0 software.

### Plasmids

The human Orc1, Orc2 and E2F1 cDNAs were obtained by RT-PCR amplification and cloned into the pcDNA3 vector (Invitrogen, USA). All the mutated and deleted versions of Orc1 were obtained by recombinant PCR and cloned into the pcDNA3 vector with the addition of an N-terminal HA tag. The vector expressing GST-E2F1 has been already described [41]. The vector expressing the 400 aa-long, GST-tagged, N-terminal fragment of Rb is a kind gift of Dr. M. Pacek [42]. The vectors expressing the GST fusion proteins containing the AB pockets of Rb, p107, and p130 [43] are a kind gift of Prof. D. Cress. The vectors expressing the GST fusion proteins of the deleted and point-mutated versions of the large ABC pocket region of Rb (AE, AB, SE, and AECys^706^Phe) [19] were kindly provided by Prof. A. Fotedar. For the FRET experiments, the E2F1, Orc1, Orc2, Mcm2, Mcm3 and Rb cDNAs were obtained by PCR and subcloned in frame in both the pEBFP-N1 and pEGFP-N1 vectors (Clontech); the Orc1-GFP, Orc2-BFP constructs have been already described [16]. All constructs were verified by nucleotide sequencing before utilization.

### GST pulldown assay

[^35^S]-labelled proteins used for in vitro binding assays were produced by using the TNT Reticulocyte Lysate System or Wheat Germ System (Promega) according to the manufacturer's instructions, by using the corresponding pcDNA3 vectors as templates. Glutahione S-transferase (GST) fusion proteins were prepared as already described [41]. GST pulldown assays were performed as already described [44]. Briefly, 1 µg of recombinant proteins, after pretreatment in a solution containing DNase I 10 mU/µl and RNase A 0.2 µg/µl to remove contaminant bacterial nucleic acids, were incubated with in vitro translated [^35^S]-proteins in a solution containing 0.1 mg/ml ethidium bromide. Following extensive washes, the reaction mixture was resolved by SDS-PAGE electrophoresis and radioactive proteins were visualized by phosphoimaging (Instantimager, Packard).

### Antibodies, immunobloting and immunoprecipitation

Polyclonal antibodies anti-ORC1 were produced and purified by immunization of rabbits with a His-tagged Orc1 250–480 Aa polypeptide [14]. Anti-Orc2 antibodies were from MBL. Monoclonal Anti-Rb (554136) antibodies were purchased from BD Biosciences/Pharmingen. Rb (IF8), Rb (C-15), E2F1 (KH95), E2F1 (C-20), Cdc6 (108.2), Cdc6 (H-304), Cyclin E (M-20), Cyclin A (H-432), Cyclin B1 (H-433), HDAC1 (C-19) and normal rabbit IgG (sc-2027) antibodies were from Santa Cruz Biotechnologies. Anti-HA (3F10) antibodies were from Roche and anti-αtubulin (B-5-1-2) antibody from Sigma. The acetyl-histone H3 (06-599) and H4 (06-866) antibodies were from Upstate. Whole cell extracts were prepared in HNNG buffer (15 mM Hepes pH 7.5, 250 mM NaCl, 1% NP-40, 5% glycerol, 1 mM PMSF) supplemented with 25 mM NaF, 10 mM β-glycerophosphate, 0.2 mM sodium orthovanadate and protease inhibitors cocktail tablet (Roche) for immunobloting. Immunoblots were carried out with 30 to 50 µg of whole-cell lysate. Immunoprecipitations were performed on 1–2 mg/ml of total protein lysate. Lysates for immunoprecipitation were incubated overnight with the appropriate amount of antibody (usually 1 to 2 µg) at 4°C. Immunocomplexes were collected with protein A/G plus agarose beads (Santa Cruz Biotechnologies), washed in HNNG buffer, treated with DNase I (Gibco BRL) for 15 min at room temperature. Beads were sequentially washed at 4°C with HLNG buffer (as HNNG but with LiCl), TE buffer and finally resuspended in Laemli sample buffer. Proteins were separated on 4–20% Tris-glycine gradient gel (Invitrogen) and detected by immunoblotting using the enhanced chemiluminescence systems (Super Signal West Dura Extended, Pierce, Rockford, IL and ECL, Amersham Bioscience).

### Chromatin immunoprecipitation assay

Cells were fixed by adding formaldehyde (Fluka) directly to the cell culture medium at 1% final concentration. Cross-linking was allowed to proceed for 7 min at 37°C and was stopped by the addition of glycine (Sigma) at a final concentration of 125 mM. Cells were washed and harvested in ice-cold PBS by centrifugation, the cellular pellet was resuspended in HNNG buffer and chromatin was sheared by sonication (average size of 0.5–1.5 kb fragments) on ice and centrifuged to pellet debris. Immunoprecipitations were performed as above. Protein-DNA immunocomplexes were collected with protein A/G plus agarose beads (Santa Cruz Biotechnologies), washed sequentially in HNNG buffer and HLNG buffer, resuspended in TE buffer and treated at 100 µg/ml of RNase A (Roche) for 30 min at 37°C. Samples were incubated for 1 h at 56°C with 0.5 mg/ml Proteinase K (Sigma), and for 15 h at 65°C to revert crosslinks. DNA was extracted with phenol:chloroform:isoamyl alcohol 25∶24∶1 (Invitrogen), ethanol precipitated and resuspended in 10 mM Tris HCl pH 7.5 for real time PCR.

### Real Time PCR

Sequence-specific primer and probe sets for real time PCR analysis were designed by Primer Express 1.5, in order to amplify and detect origin as well as non-origin areas within the human Lamin B2 and GM-CSF origins [12], [14]. Using a high resolution chroma. Real-time PCR was carried out in triplicate using Universal Master Mix (Applied Biosystems) on an ABI Prism 7000 Sequence Detector System (Applied Biosystems). Sequence Detector software (version 1.7) was utilized for data analysis. Relative fold enrichment was determined by the comparative cycle threshold (C_T_) method, detecting Ct difference between a target and a standard region of interest [45].

### FRET analysis

FRET analysis was conducted as previously described in detail [21], [46]. Briefly, HeLa cells were transiently transfected with plasmids expressing GFP and BFP fusion protein pairs by the calcium phosphate method. FRET experiments consisted in collecting two EGFP emission signals by exciting the EGFP with two different excitation wavelengths, 480 and 350 nm, the former being optimal for EGFP and the latter for BFP. The background fluorescence was measured for each field outside of the transfected cells and subtracted. The existence of FRET was inferred by determining the ratio between EGFP fluorescence following excitation at 350 nm to that following excitation at 480 nm. Confocal microscopy was performed with a Zeiss Axiovert X60 confocal microscope. Images were acquired with the LSM510 software. Data acquisition and analysis were performed using the MetaMorph software (Universal Imaging Corporation).

### RNA interference experiments

Cells were transiently transfected with smart pool siRNAs (Dharmacon) against Orc1 for 72 h at 40 nM final concentration by oligofectamine-mediated transfection (Invitrogen) following the manufacturer instructions. RNAi control experiments were performed using a duplex siRNA against luciferase (Dharmacon).

### BrdU incorporation experiments

BrdU incorporation experiments were performed on cells transiently transfected for 72 h with siRNA duplexes. Cells were pulsed for 1 h with BrdU at final concentration of 1 mM and BrdU-positive cells were detected by using a FITC conjugated anti-BrdU antibody (Abcam). Cells were collected and analyzed by double-flow cytometry analysis on a FACSCalibur (Becton Dickinson) instrument, to simultaneously determine the cell cycle profile (DNA content) by incorporation of propidium iodide, and the S phase cell population by incorporation of BrdU.

### Statistical analysis

One-way ANOVA and Bonferroni/Dunn's post-hoc test was used to compare multiple groups. Pair-wise comparison between groups was performed using the Student's t test.

## Supporting Information

Figure S1 Coomassie stained gels corresponding to the autoradiographs shown in Figure 2. At the end of the GST-pulldown experiments, samples were resolved on SDS-PAGE. Gels were then stained with Coomassie blue and dried. Radioactivity was measured after phosphoimaging (Figure 2) while gels were photographed and are shown here. GST fusion proteins are indicated by (*). Panels A-D correspond to Figure 2, panels A-D respectively; panel E to Figure 2 panel F; panel F to Figure 2 panel G.
(2.58 MB TIF)
